# Effectiveness of COVID-19 vaccine in preventing infection and disease severity: a case-control study from an Eastern State of India

**DOI:** 10.1017/S0950268821002247

**Published:** 2021-10-11

**Authors:** Chandramani Singh, Bijaya Nanda Naik, Sanjay Pandey, Bijit Biswas, Binod Kumar Pati, Manisha Verma, Prabhat Kumar Singh

**Affiliations:** 1 Department of Community and Family Medicine, All India Institute of Medical Sciences, Patna, Bihar, India; 2 Department of Microbiology, All India Institute of Medical Sciences, Patna, Bihar, India; 3 Department of Anaesthesiology and Critical Care, All India Institute of Medical Sciences, Patna, Bihar, India

**Keywords:** Case-control studies, COVID-19, effectiveness, India, SARS-CoV-2, vaccines

## Abstract

Effectiveness of corona virus disease-19 (COVID-19) vaccines used in India is unexplored and need to be substantiated. The present case-control study was planned to elicit the effectiveness of COVID-19 vaccines in preventing infection and disease severity in the general population of Bihar, India. This case-control study was conducted among people aged ≥45 years during April to June 2021. The cases were the COVID-19 patients admitted or visited All India Institute of Medical Sciences (AIIMS), Patna, Bihar, India, and were contacted directly. The controls were the individuals tested negative for severe acute respiratory syndrome coronavirus-2 (SARS CoV-2) at the Virology laboratory, AIIMS-Patna and contacted telephonically for collection of relevant information. The vaccine effectiveness (VE) was calculated by using the formula (VE = 1 – odds ratio). The adjusted VE for partial and full vaccination were estimated to be 52.0% (95% confidence interval (CI) 39.0–63.0%) and 83.0% (95% CI 73.0–89.0%) respectively for preventing SARS CoV-2 infection. The sub-group analyses of the cases have shown that the length of hospital stays (LOS) (partially vaccinated: 9 days *vs.* unvaccinated: 12 days; *P* = 0.028) and the severity of the disease (fully vaccinated: 30.3% *vs.* partially vaccinated: 51.3% and unvaccinated: 54.1%; *P* = 0.035) were significantly low among vaccinated compared to unvaccinated individuals. To conclude, four out of every five fully vaccinated individuals are estimated to be protected from contracting SARS CoV-2 infection. Vaccination lowered LOS and chances of development of severe disease.

**Research in context:**

**Evidence before this study**

We searched PubMed using the following search terms for observational studies (‘ChAdOx1 nCoV-19’ or ‘Oxford’ or ‘AstraZeneca’ or ‘BBV152’) and (‘COVID-19’ or ‘SARS-CoV-2’) and (‘vaccine’ or ‘vaccination’) and ‘effectiveness’ with no language or year restriction on 23 July 2021. We retrieved VIVALDI cohort study from the UK which reported that protection offered by a single-dose Oxford-AstraZeneca vaccine from SARS-CoV-2 infection to be 68% (at 35–48 days) among adults aged ≥65 years. Another cohort study from north-west London documented 74% risk reduction of testing positive for SARS-CoV-2 after 28 days of receiving first dose of ChAdOx1 nCoV-19. Public Health England test-negative case-control study observed vaccine effectiveness (VE) in terms of COVID-19-related symptom prevention of first dose of Oxford-AstraZeneca vaccine to be 60% between 28 and 34 days which increased to 73% after 35 days among older adults aged ≥70 years.

**Added value of this study**

Through adaptation of case-control design, we investigated the combined effectiveness of in use Covishield (Oxford-AstraZeneca) and Covaxin (Bharat Biotech) SARS-CoV-2 vaccines of India. Unadjusted combined VE was found to be 45.0% (95% CI 30.0–56.0) in the partially vaccinated group and 77.0% (95% CI 65.0–84.0%) in the fully vaccinated group in preventing SARS-CoV-2 infection. After adjusting with potential confounders, the VE for partial and full vaccination were estimated to be 52.0% (95% CI 39.0–63.0%) and 83.0% (95% CI 73.0–89.0%), respectively. We also adjusted VE with COVID-appropriate behaviour as per World Health Organisation (WHO) recommendations which other prior studies ignored. Vaccination was also found to confer protection against experiencing a severe disease and lowered hospital stay.

**Implications of all the available evidence**

This is one of the earlier evidences on real-world VE of COVID-19 vaccines used in the world's largest COVID-19 vaccination drive. Addition of the evidences generated in the present study with prior pieces of evidences available in this context will help in restoring faith regarding SARS-CoV-2 vaccination among the beneficiaries of it. This will also consolidate trust of policymakers on vaccination who issued emergency approval to these vaccines on public interest amid pandemic.

## Introduction

Vaccination is one of the safest and cost-effective public health interventions for infectious disease prevention and control, especially in a pandemic situation [1]. The corona virus disease-19 (COVID-19) pandemic which has affected 31 174 322 individuals and has claimed 414 482 lives in India as of 21 July 2021 is not an exception [2]. In the absence of a curative therapy, and the difficulty of imposing strict COVID-appropriate behaviours, the demand for safe and effective vaccine emerged early, leading to its development at an unprecedented pace. As of 15 April 2021, two COVID-19 vaccines, Covaxin and Covishield, are in use in India. The Covaxin is a virion-inactivated severe acute respiratory syndrome coronavirus-2 (SARS-CoV-2) vaccine developed and produced by Bharat Biotech whereas the Covishield is a recombinant chimpanzee adenovirus vector vaccine manufactured by Serum Institute of India (SII) with technology transfer from the Oxford University and AstraZeneca. The Covaxin, administered with two doses 4 weeks apart, is reported to have overall efficacy of 77.8%, and 93.4% against the severe symptomatic disease. The Covaxin is also reported to have good efficacy (65.2%) against the Delta (B.1.617.2) variant of SARS-CoV-2 [3]. The Covishield vaccine, also administered with two doses, claimed to have an efficacy of 55.1% for a shorter time interval (≤6 weeks) between the two doses. The increase in interval between the two doses of the Covishield vaccine to 12 weeks or more was claimed to confer 147% higher protection (vaccine efficacy: 81⋅3%) compared to the short interval [4].

India started the world's largest vaccination drive with the use of the Covaxin and the Covishield vaccine both in a phased manner against COVID-19 on 16 January 2021 [5]. In the initial two phases, only the healthcare workers (HCWs) and the front-line workers irrespective of their age were eligible for vaccination. The COVID vaccines were made available for the general public of the country in a phased manner starting from 1 March 2021 (for the elderly and the individuals aged above 45 years with the comorbidities) and from 1 May 2021 (for all adults) [6]. The interval between the doses of the Covaxin is 4 weeks and the Covishield is revised from 4 weeks at the start of the vaccination drive to 12–16 weeks as of May 2021 [7].

Both the COVID-19 vaccines in India demonstrated good clinical efficacy and received the approval from the Drug Controller of India (DGCI) for emergency use. However, the real-world effectiveness of these vaccines have been largely unexplored and not documented in Indian settings till date [3, 8–10]. Moreover, the vaccine effectiveness (VE) assessment is required to overcome the vaccine hesitancy, restoration of faith and increase the acceptance of COVID-19 vaccines among the target population to prevent and control the current pandemic [11]. As of 21 April 2021, only 8.3% and 1.3% of the total population of the country could be vaccinated with first and second doses of SARS-CoV-2 jab, respectively [12]. The case-control design is the cost-effective and rapid way of the VE assessment during an epidemic situation [10]. Previously case-control study designs have been used in the post-licencing effectiveness of the oral cholera vaccine, rota virus vaccine and the influenza vaccines [13–15]. The case-control design has already been adopted by studies from other countries for the COVID-19 VE assessment [16–18]. In this context, the present case-control study was planned to estimate the VE of COVID-19 vaccines in preventing infection and disease severity in an Eastern State of India.

## Materials and methods

We conducted an unmatched case-control study at All India Institute of Medical Sciences (AIIMS), situated in the ancient city of Patna beside the holy river Ganges between April and June 2021. All consecutive SARS CoV-2-positive patients who have availed services from our institute and the individuals who were tested negative for SARS CoV-2 at our Virology laboratory during the study period were the potential cases and controls for the study, respectively. We considered only the first visit of the cases for enrolment. Notably, none of our recruited cases were reinfected during the study period which was one of the pre-set exclusion criteria. In either group, only one participant from a family having multiple eligible participants (index-one) was considered. The controls with a history of COVID-19 or influenza-like illness (ILI) in the preceding 3 months from the day of data collection were excluded. The Institutional Ethical Committee (IEC) of AIIMS, Patna (Ref. AIIMS/Pat/IEC/2020/706) approved the study protocol.

The odds ratio (OR) of contracting infection following COVID-19 vaccination was calculated to be 0.3 with the anticipated VE of 70%. During April 2021, the first dose COVID-19 vaccination coverage among the eligible general population of Bihar was found to be 3.7%. Considering the vaccination coverage (3.7%) and OR (0.3), the ratio of controls to cases 2:1, 95% precision and 80% power, the minimum sample size for the study was calculated to be 507 cases and 1013 controls using the online software OpenEpi [12, 19, 20]. A total of 577 cases and 1154 controls were selected during the study period and considered for the final analyses. The post-hoc power analysis using OpenEpi software showed the adequacy of final sample size in our study (full vaccination (cases *vs.* control: 5.7% *vs.* 17.4%; power: 100.0%); partial vaccination (cases *vs.* control: 26.7% *vs.* 34.3%; power: 88.8%)) [21].

**Fig. 1.**
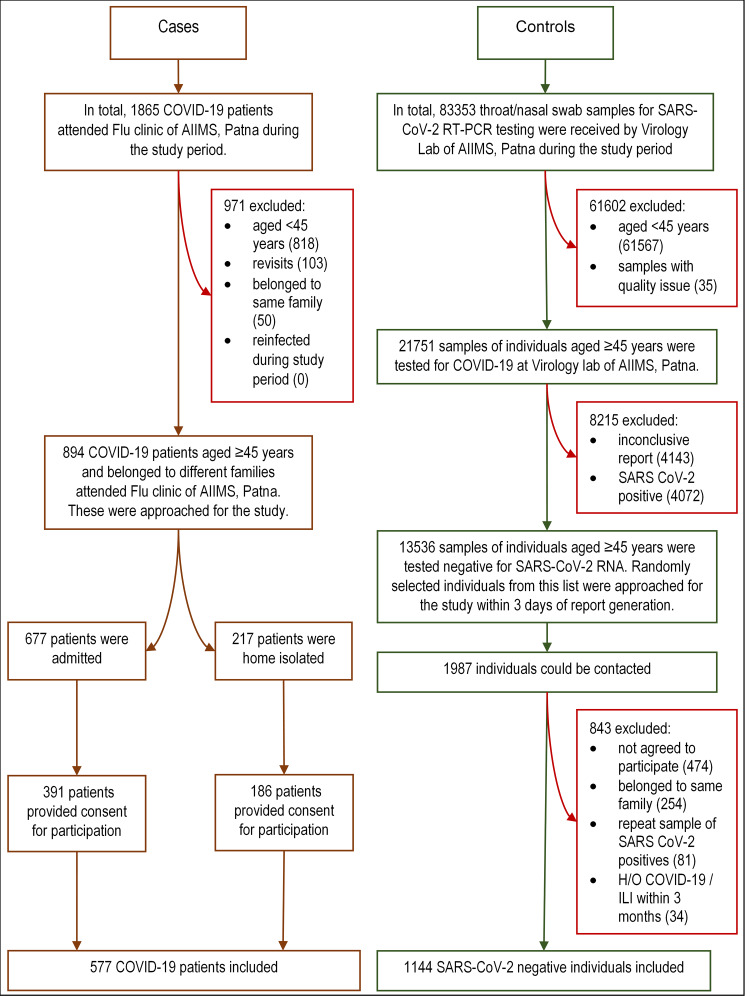
Flowchart showing recruitment of the cases and controls. COVID-19, corona virus disease-19; RT-PCR, reverse transcriptase polymerase chain reaction; SARS-CoV-2, severe acute respiratory syndrome coronavirus-2; RNA, ribonucleic acid; H/O, history of; ILI: influenza-like illness.

We approached consecutive COVID-19 patients aged ≥45 years (as during protocol development vaccination for 18–44 had not started) with a valid report (rapid antigen (Ag) or reverse transcriptase polymerase chain reaction (RT-PCR) for SARS-CoV-2) who have attained COVID dedicated flu clinic of our institute during the study period. Consented individuals were included as cases and were interviewed face-to-face. The information for severe and moribund cases was obtained from accompanying family members or friends directly. The highest disease severity of hospitalised cases was later retrieved from their medical records, while for home isolated ones, it was inquired telephonically. Line lists of RT-PCR-negative individuals aged ≥45 years were collected on a daily basis from our Virology laboratory during the study period. We telephonically approached individuals from these line lists at random within 3 days of their report and the consenting individuals were included as controls. All interviews were done by trained resident doctors of our institute. Details of case and control recruitment process are depicted in Fig. 1.

The study schedule was largely adopted from the World Health Organization (WHO) guidelines on the evaluation of COVID-19 effectiveness (2021) and modified in the local context [10]. The final study schedule comprised of age, sex, occupation, chronic co-morbidity status, history of (H/O) hospitalisation for chronic co-morbidities in past 5 years, COVID-19 vaccination profile (vaccination status; name of the vaccine, number of doses, date of first dose, date of second dose; the predominant reason for not getting vaccinated), symptom profile, H/O ILI, past COVID-19 infection and high-risk contact with COVID-19 case or suspect since March 2020, COVID-appropriate behaviour profile of last 14 days (use of mask while going outdoors, adherence to social distancing, avoidance of crowded places, handwashing before touching face); date of RT-PCR/rapid Ag test; hospitalisation status and disease characteristics (for cases only) (disease severity, length of hospital stay (LOS) and final treatment outcome).

### Operational definitions

Severity of COVID-19, **Mild:** No complaints of breathlessness and normal pulse oxygen saturation (SpO_2_): ≥94% and respiratory rate (RR): <24 breaths per minute (bpm); **Moderate:** Complaint of breathlessness and/or SpO2: 90–93% on room air and/or RR: 24–30 bpm with no features of severe disease (i.e. shock, organ dysfunction); **Severe:** SpO2: <90% on room air and/or RR: >30 bpm and/or features of severe disease (i.e. shock, organ dysfunction) [22, 23]. Four items of the COVID-appropriate behaviour were scored as follows: always (0), sometimes (1) and never (2). Scores of these four items were summed-up to obtain the total score where a higher score indicated more COVID-inappropriate behaviour. The definitions used for ascertaining COVID-19 vaccination status were as follows, **unvaccinated:** At the time of testing for SARS-CoV-2, one has not received jab or was within 14 days of first jab; **partially vaccinated:** did the testing for SARS-CoV-2, ≥14 days of first jab or before 14 days of second jab; **fully vaccinated:** did the testing for SARS-CoV-2, ≥14 days after second jab.

### Statistical analysis

The information collected was entered using Epicollect5 (an open-source data collection software) [24]. The data entered were downloaded and imported to statistical package for social sciences (SPSS) (version 22.0) for analysis. The quantitative variables were expressed as median (interquartile range (IQR)). The categorical variables were expressed as proportion and percentages. The Mann–Whitney *U* test and Kruskal–Wallis test were used to compare medians across binomial and ordinal variables, respectively. Bonferroni's test was used for post-hoc analysis of ordinal variables. Pearson's *χ*^2^ test was used to test the association between categorical variables. The *χ*^2^ adjusted standardised test was used for higher order contingency table (>2 × 2) to identify significant pair. The protective effect of COVID vaccines for contracting COVID-19 infection was statistically tested using univariable logistic regression followed by multivariable logistic regression, and quantification was expressed as OR. We adjusted for all actual or potential confounders (age, sex, occupation, COVID-inappropriate behaviour score, chronic co-morbidity, H/O hospitalisation, ILI, prior COVID-19 and high-risk contact with a case or suspect) in the multivariable logistic regression model. Unadjusted and adjusted VE were calculated using the following formulae: (VE = (1 – OR) × 100%). We used 95% confidence level for all the statistical tests used.

## Result

The median age of the cases was higher than the controls while male gender constituted about two-third of the study population in both the groups. About three-fifth (58.8%) of the cases and one-fourth of the controls (28.8%) were having at least one chronic co-morbidity. About two-fifth of the cases (42.1%) and three-fifth of the controls (61.4%) have received at least a single jab. About two-third and one-fifth of the study participants in both the groups have received Covishield and Covaxin, respectively (Table 1).

**Table 1.** Background characteristics of the study participants

Variable	Cases *N* = 577, *N* [%]	Controls *N* = 1154, *N* [%]	*P*-value
Age in years: median {IQR}	59 {52–67}	53 {48–60}	<0.001^a^
Sex			
Male	410 [71.1]	743 [64.4]	0.006^b^
Female	167 [28.9]	411 [35.6]	
Occupation: HCW	38 [6.6]	61 [5.3]	0.272^b^
Chronic co-morbidity	335 [58.1]	332 [28.8]	<0.001^b^
Hypertension	236 [70.4]	237 [71.4]	
Diabetes	171 [51.0]	148 [44.6]	
CVD	51 [15.2]	31 [9.3]	
COPD/Asthma	23 [6.9]	19 [5.7]	
Hypothyroidism	30 [9.0]	11 [3.3]	
CKD	9 [2.7]	8 [2.4]	
Cancer	5 [1.5]	9 [2.7]	
TB	10 [3.0]	7 [2.1]	
Other chronic co-morbidity*	5 [1.5]	2 [0.6]	
H/O hospitalisation for co-morbidities	52 [9.0]	23 [2.0]	<0.001^b^
Symptomatic on arrival	474 [82.1]	56 [4.9]	<0.001^b^
Fever	325 [68.6]	46 [82.1]	
Cough	327 [69.0]	37 [66.1]	
Breathlessness	283 [59.7]	6 [10.7]	
Sore throat	85 [17.9]	20 [35.7]	
Diarrhoea	29 [6.1]	2 [3.6]	
Chest pain	36 [7.6]	1 [1.8]	
Malaise	117 [24.7]	20 [35.7]	
Loss of taste	55 [11.6]	0 [0.0]	
Loss of smell	40 [8.4]	1 [1.8]	
Other symptoms^#^	18 [3.8]	0 [0.0]	
H/O ILI	59 [10.2]	128 [11.1]	0.584^b^
H/O past COVID-19 infection	15 [2.6]	101 [8.8]	<0.001^b^
COVID-inappropriate behaviour score: **median {IQR}	2 {1–4}	2 {0–3}	<0.001^a^
H/O high-risk contact with COVID-19 case or suspect	141 [24.4]	124 [10.7]	<0.001^b^
Received COVD-19 vaccination	243 [42.1]	709 [61.4]	<0.001^b^
Type of vaccine received			
Covishield	162 [66.7]	478 [67.4]	0.667^b^
Covaxin	48 [19.8]	124 [17.5]	
Cannot recall	33 [13.6]	107 [15.1]	
Number of vaccination doses received			
One	194 [79.8]	412 [58.1]	<0.001^b^
Two	49 [20.2]	297 [41.9]	
Duration between first vaccine dose and COVID testing date in days: median {IQR}	27 {14–45}	40 {23–62}	<0.001^a^
Duration between second vaccine dose and COVID testing date in days: median {IQR}	23 {12–48}	24 {10–48}	<0.001^a^

HCW, health care worker; CVD, cardiovascular disease; COPD, chronic obstructive pulmonary disease; CKD, chronic kidney disease; TB, tuberculosis; H/O, history of; COVID-19, corona virus disease-19; ILI, influenza-like illness; IQR, interquartile range; RTPCR, reverse transcriptase polymerase chain reaction.

a Independent samples Mann–Whitney *U* test.

b *χ*^2^ test.

*Other chronic co-morbidities include stroke: 3; liver disease: 2; sarcoidosis: 1; epilepsy: 1; ^#^Other symptoms include headache: 6; swelling in eye: 5; loss of appetite: 4; haemoptysis: 2; nausea/vomiting: 2. **It was calculated based on the use of mask while going outdoors, adherence to social distancing, avoidance of crowded places, handwashing before touching face in preceding 14 days of COVID testing. { }: interquartile range; [ ]: column percentage.

**Fig. 2.**
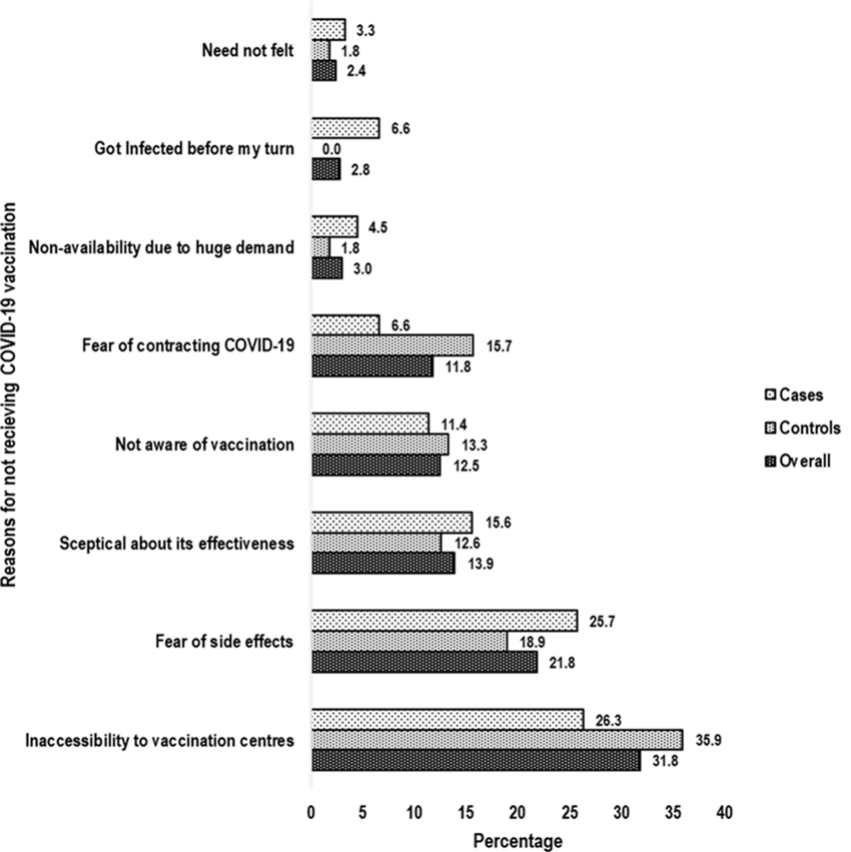
Bar chart showing predominant reasons for not receiving COVID-19 vaccination. COVID-19, corona virus disease-19.

Inaccessibility to vaccination centres (overall: 31.8%; cases: 26.3%, controls: 35.9%) was reported to be the predominant reason for not getting vaccinated followed by fear of side effects (overall: 21.8%; cases: 25.7%, controls: 18.9%) and sceptical about VE (overall: 13.9%; case: 15.6%, controls: 12.6%) (Fig. 2).

Unadjusted VE was found to be 45.0% (95% CI 30.0–56.0) in the partially vaccinated group and 77.0% (95% CI 65.0%–84.0%) in the fully vaccinated group in preventing SARS-CoV-2 infection. After adjusting with potential confounders like age, sex, occupation, COVID-inappropriate behaviour score, chronic co-morbidity, H/O hospitalisation for chronic co-morbidity, ILI, past COVID-19 infection and high-risk contact with a COVID-19 case or suspect, the VE for partial and full vaccination were estimated to be 52.0% (95% CI 39.0–63.0%) and 83.0% (95% CI 73.0–89.0%), respectively. A full vaccination course was calculated to have provided 31.0% additional protection compared to partial vaccination in preventing SARS-CoV-2 infection (Table 2).

**Table 2.** Vaccine effectiveness in preventing COVID-19 infection

Variables	Total	COVID-19 infection		Unadjusted odds ratio, OR (95% CI)	Unadjusted vaccine effectiveness % (95% CI)	Adjusted odds ratio, OR (95% CI)	Adjusted vaccine effectiveness % (95% CI)
	*N* = 1731, *N* [%]	Yes, *N* = 577, *N* (%)	No, *N* = 1154, *N* (%)				
Age in completed years: median {IQR}	55 {50–63}	59 {52–67}	53 {48–60}	1.05 (1.04–1.07)	–	1.05 (1.04–1.06)	–
Gender: males	1153 [66.6]	410 (35.6)	743 (64.4)	1.36 (1.09–1.69)	–	1.36 (1.06–1.73)	–
Occupation: HCW	99 [5.7]	38 (38.4)	61 (61.6)	1.26 (0.83–1.92)	–	1.87 (1.11–3.14)	–
Chronic co-morbidity: yes	667 [38.5]	335 (50.2)	332 (49.8)	3.43 (2.78–4.22)	–	2.51 (1.97–3.19)	–
H/O hospitalisation for co-morbidities: yes	75 [4.3]	52 (69.3)	23 (30.7)	4.87 (2.95–8.04)	–	3.11 (1.74–5.57)	–
H/O ILI: no	1544 [89.2]	518 (33.5)	1026 (66.5)	1.09 (0.79–1.52)	–	1.08 (0.72–1.62)	–
H/O past COVID-19 infection: no	1615 [93.3]	562 (34.8)	1053 (65.2)	3.59 (2.07–6.24)	–	5.33 (2.83–10.04)	–
COVID-inappropriate behaviour score: ^a^median {IQR}	2 {0–4}	2 {1–4}	2 {0–3}	1.15 (1.09–1.20)	–	1.12 (1.05–1.18)	–
H/O high-risk contact with COVID-19 case or suspect: yes	265 [15.3]	141 (53.2)	124 (46.8)	2.69 (2.06–3.50)	–	3.86 (2.80–5.31)	–
Vaccination status^b^							
Partially vaccinated	550 [31.8]	154 (28.0)	396 (72.0)	0.55 (0.44–0.70)	45.0 (30.0–56.0)	0.48 (0.37–0.61)	52.0 (39.0–63.0)
Fully vaccinated	234 [13.5]	33 (14.1)	201 (85.9)	0.23 (0.16–0.35)	77.0 (65.0–84.0)	0.17 (0.11–0.27)	83.0 (73.0–89.0)

COVID-19, corona virus disease-19; IQR, interquartile range; HCW, health care worker; H/O, history of; ILI, influenza-like illness; OR, odds ratio; CI, confidence interval. Bivariate and multivariable logistic regression was done to calculate OR.

a It was calculated based on the use of mask while going outdoors, adherence to social distancing, avoidance of crowded places, handwashing before touching face in preceding 14 days of COVID testing.

b The definitions used for ascertaining COVID-19 vaccination status were as follows, unvaccinated: at the time of testing for SARS-CoV-2, one has not received jab or was within 14 days of first jab; partially vaccinated: did the testing for SARS-CoV-2, ≥14 days of first jab or before 14 days of second jab; fully vaccinated: did the testing for SARS-CoV-2, ≥14 days after second jab. { }: interquartile range; [ ]: column percentage; (): row percentage. The vaccine effectiveness of the Covishield and Covaxin subgroups is being reported in Supplementary Tables S1 and S2 respectively.

The difference in median LOS among the cases between partially vaccinated (9 days (IQR 5–13 days)) and unvaccinated (12 days (IQR 6–16 days)) was statistically significant (*P* = 0.028). The fully vaccinated COVID patients had a lower probability of having the severe disease on arrival (18.2%) compared to the partially vaccinated (31.8%) and the unvaccinated (42.6%) cases (*P* = 0.022). Overall, the fully vaccinated COVID patients were also found to have less likelihood of experiencing severe disease (30.3%) during their illness compared to partially vaccinated (51.3%) and unvaccinated (54.1%) cases (*P* = 0.035). The final outcome (death/recovery) was statistically indifferent across the different vaccination groups (Table 3).

**Table 3.** Effect of COVID-19 vaccination on disease characteristics of the cases

Variable	Total, *N* [%]	Symptomatic, *N* (%)	Hospitalised, *N* (%)	LOS median {IQR}	Disease severity on arrival			Highest disease severity			Final outcome	
					Mild, *N* (%)	Moderate, *N* (%)	Severe, *N* (%)	Mild, *N* (%)	Moderate, *N* (%)	Severe, *N* (%)	Death, *N* (%)	Recovery, *N* (%)
Vaccination status*												
Unvaccinated	390 [67.6]	320 (82.1)	260 (66.7)	12 {6–16}	169 (43.3)	55 (14.1)	166 (42.6)	136 (34.9)	43 (11.0)	211 (54.1)	110 (28.2)	280 (71.8)
Partially vaccinated	154 [26.7]	128 (83.1)	111 (72.1)	9 {5–13}	79 (51.3)	26 (16.9)	49 (31.8)	54 (35.1)	21 (13.6)	79 (51.3)	33 (21.4)	121 (78.6)
Fully vaccinated	33 [5.7]	26 (78.8)	20 (60.6)	10 {6–15}	20 (60.6)	7 (21.2)	6 (18.2)	14 (42.4)	9 (27.3)	10 (30.3)	6 (18.2)	27 (81.8)
***P*-value**		0.837^a^	0.316^a^	0.034^b,†^		0.022^a,‡^			0.035^a,§^		0.156^a^	

LOS, length of hospital stay in days; IQR, interquartile range. ^a^*χ*^2^ test; ^b^Kruskal–Wallis test. *The definitions used for ascertaining COVID-19 vaccination status were as follows, unvaccinated: at the time of testing for SARS-CoV-2, one has not received jab or was within 14 days of first jab; partially vaccinated: did the testing for SARS-CoV-2, ≥14 days of first jab or before 14 days of second jab; fully vaccinated: did the testing for SARS-CoV-2, ≥14 days after second jab. ^†^In Bonferroni post-hoc test, difference in median LOS between partially vaccinated and unvaccinated (*P* = 0.028) was significantly different. ^‡^In post-hoc analysis, using adjusted standardised proportion of severe disease was significantly lower in fully vaccinated group compared to others while this was significantly higher in unvaccinated compared to others. ^§^In post-hoc analysis, using adjusted standardised proportion of severe disease was significantly lower in fully vaccinated compared to others. [ ]: column percentage; (): row percentage; { }: interquartile range. The disease characteristics of the Covishield and Covaxin subgroups are being reported in Supplementary Tables S3 and S4 respectively.

VE and disease characteristics of Covaxin and Covishield sub-groups are presented in Supplementary Tables S1–S4.

## Discussion

The Government of India is rolling out the emergency use of two COVID vaccines in an unprecedented manner to control the ongoing pandemic, which is now open to all adults of the country. Still, the coverage of vaccination is less despite 6 months into the vaccination campaign. One of the crucial reasons is the sceptical attitude of public towards the protection offered by the in use COVID-19 vaccines. Till date, the real-world effectiveness of the COVID vaccines used in India is not documented. We generated the evidence on the effectiveness of COVID-19 vaccines used in India through this study. We found the partial vaccination to be 52% effective in preventing a SARS-CoV-2 infection and that the two doses to be 83% effective. The COVID-19 vaccination was found to reduce the LOS and likelihood of severe disease among cases.

We found the single dose of COVID-19 vaccines to be 52.0% effective which was low compared to the efficacy of 76.0% claimed by Oxford-AstraZeneca excluding the initial 21 days post-vaccination [4]. VIVALDI cohort study [25], which requited older adults (≥65 years) of the UK as study participants, reported 68% risk reduction (at 35–48 days) of contracting SARS-CoV-2 following a single jab of Oxford-AstraZeneca vaccine. Even higher risk reduction of infection (74% (28 days onwards)) following a single standard dose of Oxford-AstraZeneca vaccine was claimed by Glampson *et al*. [26] which enrolled participants of all ages (≥16 years) in their study. Both these observations were higher compared to us. Higher single-dose effectiveness of Oxford-AstraZeneca vaccine has also been reported by Lopez Bernal *et al*. [17] (60% (between 28 and 34 days), 73% (post 35 days)) from the UK using a test-negative case-control design among older adults (≥70 years). There could be multiple reasons for the differences in the VE results in our study and the studies reported from the UK. The foremost reason is the age of the study participants. The chance of getting infection and having COVID-19 increases with age, so also the VE [27, 28]. The participants in all the UK studies were comparatively older than our study except for the study reported from north-west London [26]. Lopez Bernal *et al*. [17] examined VE for disease prevention by contrast we checked VE for infection prevention. Generally, the VE of disease prevention is higher than the VE of infection prevention [29]. Second, unlike us, Shrotri *et al*. [25] and Glampson *et al*. [26] adopted a cohort study design for VE assessment. Moreover, ethnic variations might have played a role. Lastly, all the UK studies were done during December 2020 to March 2021 when the predominant viral strain circulating in that country was B.1.1.7 [30]. During our survey, the predominant SARS-CoV-2 variant circulating in our country was B.1.617 [31]. Viral strain variation is a known influencer of VE [29]. We found the effectiveness of 83.0% after full vaccination in India. The vaccine efficacy (81.3%) reported by Oxford-AstraZeneca with two standard doses at ≥12 weeks interval was similar to our observations [4]. The Covaxin phase III trial has reported vaccine efficacy of 77.8% in the prevention of symptomatic COVID-19 which was also in line with our observations [3]. We claim our results to be more accurate, not only due to adjustment for the potential and actual confounders, but also the inclusion of ‘COVID-appropriate behaviour following vaccination’ in the confounder list which others have omitted due to varied reasons.

The predominant reason for not receiving vaccine against COVID-19 was reported to be the inaccessibility to the vaccination centre. Online registration through CoWIN app is mandatory to get vaccine in India. Signal cloud congestion along with the necessity for prior appointment to get the vaccine restricted the accessibility to the vaccination centre. Above that the low literacy in Bihar [19] would have contributed towards the poor understanding and operations of CoWIN App among the beneficiaries. The second and third most common reasons behind non-vaccination were the fear of side effects and sceptical about VE. The findings of this study can be used to establish the trust of common people and help improve the vaccination coverage in the fight against the COVID-19.

### Strengths and limitations

This is one of the earlier evidences on the VE of COVID-19 vaccines used in India. Despite an extensive literature search, we could not come across a published piece of literature on the VE of COVID vaccines used in India. We have recruited two controls for each case which has increased the power of the study. The post-hoc power analysis revealed that the study had 100.0% and 88.8% power to elicit the differences between cases and controls in terms of full and partial vaccination, respectively. The controls recruited were RT-PCR-negative for SARS-CoV-2 ribonucleic acid (RNA). We have also adjusted for the confounders in the final analysis. Most importantly, we have adjusted for the COVID-19-appropriate behaviour in our study.

Like any other observational study, our study also has few limitations. First, we recruited cases only from one centre, AIIMS, Patna which is the highest referral institute for the management of COVID-19 in Bihar. Patients from all the corners of the state are being referred here. Thus, the cases of the present study might represent COVID-19 cases for the state of Bihar to a large extent. Second, the telephonic interview was used as a mode of data collection for home-isolated cases and controls. Inherent biases associated with the telephonic interview might be present. The telephonic interviews were made by trained resident doctors, and repeat calls following consent were made in case of any doubt over a piece of information. This might have alleviated the bias to some extent. Third, the errors associated with self-reporting of some information. We tried to reduce these errors by developing rapport with the participant and allowing sufficient time to respond. We also requested them to confirm the vaccination details by cross-checking with the CoWIN app or the message received following vaccination. Fourth, the misclassification due to the inherent properties of the test used for the investigation of SARS-CoV-2. The RT-PCR-based testing is only 60–70% sensitive, although specificity is claimed to be more than 90%. Lastly, we did not have a sufficient sample size to do sub-group analysis for Covishield and Covaxin groups. However, the analysis and findings with the samples at our hand are provided in Supplementary Tables.

## Conclusions

COVID-19 vaccination was found to be effective in infection prevention. One out of two and four out of five individuals were found to be protected against SARS-CoV-2 infection following partial and full vaccination, respectively. The vaccinated individuals had lesser LOS compared to unvaccinated ones. Additionally, the fully vaccinated individuals were less likely to develop severe disease. We recommend studies with a larger sample size to elicit the VE of individual COVID-19 vaccines.

## Data Availability

All data generated or analysed during this study are included in this published article (and its Supplementary information files).

## Supplementary material

For supplementary material accompanying this paper visit http://dx.doi.org/10.1017/S0950268821002247. click here to view supplementary material
