# Supplementary material for: Effectiveness of COVID-19 vaccine in preventing infection and disease severity: a case-control study from an Eastern State of India
Source: Epidemiol Infect. 2021 Oct 11;149:e224. doi: 10.1017/S0950268821002247 (PMC8545845; doi:10.1017/S0950268821002247)
Supplement: Supplementary file 1 [file S0950268821002247sup001.docx]

**Supplementary Table 1. Effectiveness of Covishield in Preventing COVID-19 Infection**

| **Variables** | **Total** | **COVID-19 Infection** | | **Unadjusted Odds Ratio**  **OR (95% CI)** | **Unadjusted Vaccine Effectiveness**  **% (95% CI)** | **Adjusted**  **Odds Ratio**  **OR (95% CI)** | **Adjusted Vaccine Effectiveness**  **% (95% CI)** |
| --- | --- | --- | --- | --- | --- | --- | --- |
|  | **N=1419**  **N [%]** | **Yes**  **N=496**  **N (%)** | **No**  **N=923**  **N (%)** |  |  |  |  |
| **Age in completed years:** Median {IQR} | 55 {50-63} | 59 {52-67} | 53 {48-60} | 1.06 (1.05, 1.07) | - | 1.05 (1.04, 1.06) | - |
| **Gender:** Males | 946 [66.7] | 349 (36.9) | 597 (63.1) | 1.30 (1.02, 1.64) | - | 1.33 (1.02, 1.74) | - |
| **Occupation:** HCW | 82 [5.8] | 26 (31.7) | 56 (68.3) | 0.86 (0.53, 1.38) | - | 1.43 (0.79, 2.57) | - |
| **Chronic co-morbidity:** Yes | 552 [38.9] | 283 (51.3) | 269 (48.7) | 3.23 (2.57, 4.05) | - | 2.42 (1.86, 3.14) | - |
| **H/O Hospitalisation for co-morbidities:** Yes | 61 [4.3] | 44 (72.1) | 17 (27.9) | 5.19 (2.93, 9.18) | - | 3.58 (1.81, 7.08) | - |
| **H/O ILI:** No | 1253 [88.3] | 443 (35.4) | 810 (64.6) | 1.17 (0.82, 1.65) | - | 0.99 (0.65, 1.54) | - |
| **H/O Past COVID-19 Infection:** No | 1320 [93.0] | 483 (36.6) | 837 (63.4) | 3.82 (2.11, 6.91) | - | 5.48 (2.76, 10.88) | - |
| **COVID inappropriate behaviour score:^*^** Median {IQR} | 2 {0-4} | 2 {1-4} | 2 {0-3} | 1.18 (1.12, 1.24) | - | 1.14 (1.07, 1.21) | - |
| **H/O high risk contact with COVID-19 case or suspect:** Yes | 226 [15.9] | 119 (52.7) | 107 (47.3) | 2.41 (1.80, 3.21) | - | 3.59 (2.54, 5.08) | - |
| **Vaccination Status:^#^** |  |  |  |  |  |  |  |
| Partially Vaccinated | 383 [27.0] | 109 (28.5) | 274 (71.5) | 0.56 (0.43, 0.72) | 44.0 (28.0, 57.0) | 0.51 (0.38, 0.68) | 49.0 (32.0, 62.0) |
| Fully Vaccinated | 141 [9.9] | 15 (10.6) | 126 (89.4) | 0.17 (0.10, 0.29) | 83.0 (71.0, 90.0) | 0.12 (0.06, 0.21) | 88.0 (79.0, 94.0) |
| COVID-19: corona virus disease-19; IQR: interquartile range; HCW: health care worker; H/O: history of; ILI: influenza like illness; OR: odds ratio; CI = Confidence interval. Bivariate and multivariable logistic regression was done to calculate OR. ^*^It was calculated based on use of mask while going outdoors, adherence to social distancing, avoidance of crowded places, handwashing before touching face in preceding 14 days of COVID testing. ^#^The definitions used for ascertaining COVID-19 vaccination status were as follows, unvaccinated: At the time of testing for SARS-CoV-2, one has not received jab or was within 14 days of first jab; partially vaccinated: did the testing for SARS-CoV-2, ≥14 days of first jab or before 14 days of second jab; fully vaccinated: did the testing for SARS-CoV-2, ≥14 days after second jab. { }: interquartile range; . [ ]: column percentage; ( ): row percentage. | | | | | | | |

**Supplementary Table 2. Effectiveness of Covaxin in Preventing COVID-19 Infection**

| **Variables** | **Total** | **COVID-19 Infection** | | **Unadjusted Odds Ratio**  **OR (95% CI)** | **Unadjusted Vaccine Effectiveness**  **% (95% CI)** | **Adjusted**  **Odds Ratio**  **OR (95% CI)** | **Adjusted Vaccine Effectiveness**  **% (95% CI)** |
| --- | --- | --- | --- | --- | --- | --- | --- |
|  | **N=951**  **N [%]** | **Yes**  **N=382**  **N (%)** | **No**  **N=569**  **N (%)** |  |  |  |  |
| **Age in completed years:** Median {IQR} | 55 {49-64} | 59 {51-67} | 53 {47-60} | 1.05 (1.04, 1.07) | - | 1.05 (1.03, 1.06) | - |
| **Gender:** Males | 627 [65.9] | 262 (41.8) | 365 (58.2) | 1.22 (0.93, 1.61) | - | 1.28 (0.93, 1.75) | - |
| **Occupation:** HCW | 36 [3.8] | 19 (52.8) | 17 (47.2) | 1.70 (0.87, 3.31) | - | 1.87 (0.84, 4.20) | - |
| **Chronic co-morbidity:** Yes | 382 [40.2] | 224 (58.6) | 158 (41.4) | 3.69 (2.80, 4.85) | - | 2.69 (1.97, 3.67) | - |
| **H/O Hospitalisation for co-morbidities:** Yes | 53 [5.6] | 41 (77.4) | 12 (22.6) | 5.58 (2.89, 10.77) | - | 3.62 (1.71, 7.69) | - |
| **H/O ILI:** No | 860 [90.4] | 345 (40.1) | 515 (59.9) | 0.98 (0.63, 1.52) | - | 1.08 (0.62, 1.86) | - |
| **H/O Past COVID-19 Infection:** No | 890 [93.6] | 372 (41.8) | 518 (58.2) | 3.66 (1.84, 7.31) | - | 5.52 (2.50, 12.21) | - |
| **COVID inappropriate behaviour score:^*^** Median {IQR} | 2 {0-4} | 3 {1-4} | 2 {0-4} | 1.18 (1.11, 1.27) | - | 1.18 (1.10, 1.27) | - |
| **H/O high risk contact with COVID-19 case or suspect:** Yes | 145 [15.2] | 89 (61.4) | 56 (38.6) | 2.78 (1.93, 4.00) | - | 3.82 (2.46, 5.92) | - |
| **Vaccination Status:^#^** |  |  |  |  |  |  |  |
| Partially Vaccinated | 85 [8.9] | 28 (32.9) | 57 (67.1) | 0.66 (0.41, 1.06) | 44.0 (-6.0, 59.0) | 0.55 (0.32, 0.94) | 45.0 (6.0, 68.0) |
| Fully Vaccinated | 67 [7.0] | 13 (19.4) | 54 (80.6) | 0.32 (0.17, 0.60) | 68.0 (40.0, 83.0) | 0.29 (0.15, 0.59) | 71.0 (41.0, 85.0) |
| COVID-19: corona virus disease-19; IQR: interquartile range; HCW: health care worker; H/O: history of; ILI: influenza like illness; OR: odds ratio; CI = Confidence interval. Bivariate and multivariable logistic regression was done to calculate OR. ^*^It was calculated based on use of mask while going outdoors, adherence to social distancing, avoidance of crowded places, handwashing before touching face in preceding 14 days of COVID testing. ^#^The definitions used for ascertaining COVID-19 vaccination status were as follows, unvaccinated: At the time of testing for SARS-CoV-2, one has not received jab or was within 14 days of first jab; partially vaccinated: did the testing for SARS-CoV-2, ≥14 days of first jab or before 14 days of second jab; fully vaccinated: did the testing for SARS-CoV-2, ≥14 days after second jab. { }: interquartile range; . [ ]: column percentage; ( ): row percentage. | | | | | | | |

**Supplementary Table 3. Effect of COVID-19 Vaccination on Disease Characteristics of the Cases in the Covishield Subgroup**

| **Variable** | **Total**  **N [%]** | **Symptomatic**  **N (%)** | **Hospitalised**  **N (%)** | **LOS**  **Median {IQR}** | **Disease severity on arrival:** | | | **Highest disease severity:** | | | **Final Outcome:** | |
| --- | --- | --- | --- | --- | --- | --- | --- | --- | --- | --- | --- | --- |
|  |  |  |  |  | **Mild**  **N (%)** | **Moderate**  **N (%)** | **Severe**  **N (%)** | **Mild**  **N (%)** | **Moderate**  **N (%)** | **Severe**  **N (%)** | **Death**  **N (%)** | **Recovery**  **N (%)** |
| **Vaccination status:^*^** |  |  |  |  |  |  |  |  |  |  |  |  |
| Unvaccinated | 372 [75.0] | 308 (82.8) | 250 (67.2) | 12 {6-16} | 161 (43.3) | 52 (14.0) | 159 (42.7) | 128 (34.4) | 42 (11.3) | 202 (54.3) | 107 (28.8) | 265 (71.2) |
| Partially Vaccinated | 109 [22.0] | 90 (82.6) | 78 (71.6) | 10 {6-14} | 59 (54.1) | 14 (12.8) | 36 (33.0) | 40 (36.7) | 15 (13.8) | 54 (49.5) | 20 (18.3) | 89 (81.7) |
| Fully Vaccinated | 15 [3.0] | 15 (100.0) | 12 (80.0) | 12 {7-23} | 7 (46.7) | 4 (26.7) | 4 (26.7) | 3 (20.0) | 4 (26.7) | 8 (53.3) | 4 (26.7) | 11 (73.3) |
| **p-value** |  | 0.211^a^ | 0.431^a^ | 0.182^b^ |  | 0.155^a^ |  |  | 0.349^a^ |  | 0.095^a^ | |

LOS: length of hospital stay in days; IQR: interquartile range.  ^a^Chi-square test; ^b^Kruskal-Wallis Test. ^*^The definitions used for ascertaining COVID-19 vaccination status were as follows, unvaccinated: At the time of testing for SARS-CoV-2, one has not received jab or was within 14 days of first jab; partially vaccinated: did the testing for SARS-CoV-2, ≥14 days of first jab or before 14 days of second jab; fully vaccinated: did the testing for SARS-CoV-2, ≥14 days after second jab. [ ]: column percentage; ( ): row percentage; { }: interquartile range.

**Supplementary Table 4. Effect of COVID-19 Vaccination on Disease Characteristics of the Cases in the Covaxin Subgroup**

| **Variable** | **Total**  **N [%]** | **Symptomatic**  **N (%)** | **Hospitalised**  **N (%)** | **LOS**  **Median {IQR}** | **Disease severity on arrival:** | | | **Highest disease severity:** | | | **Final Outcome:** | |
| --- | --- | --- | --- | --- | --- | --- | --- | --- | --- | --- | --- | --- |
|  |  |  |  |  | **Mild**  **N (%)** | **Moderate**  **N (%)** | **Severe**  **N (%)** | **Mild**  **N (%)** | **Moderate**  **N (%)** | **Severe**  **N (%)** | **Death**  **N (%)** | **Recovery**  **N (%)** |
| **Vaccination status:^*^** |  |  |  |  |  |  |  |  |  |  |  |  |
| Unvaccinated | 341 [89.3] | 289 (84.8) | 231 (67.7) | 12 {6-16} | 144 (42.2) | 47 (13.8) | 150 (44.0) | 116 (34.0) | 36 (10.6) | 189 (55.4) | 102 (29.9) | 239 (70.1) |
| Partially Vaccinated | 28 [7.3] | 22 (78.6) | 23 (82.1) | 11 {4-15} | 12 (42.9) | 8 (28.6) | 8 (28.6) | 7 (25.0) | 5 (17.9) | 16 (57.1) | 8 (28.6) | 20 (71.4) |
| Fully Vaccinated | 13 [3.4] | 10 (76.9) | 7 (53.8) | 9 {4-11} | 9 (69.2) | 2 (15.4) | 2 (15.4) | 7 (53.8) | 4 (30.8) | 2 (15.4) | 2 (15.4) | 11 (84.6) |
| **p-value** |  | 0.537^a^ | 0.151^a^ | 0.111^b^ |  | 0.047^a **^ |  |  | 0.024^a ††^ |  | 0.526^a^ | |

LOS: length of hospital stay in days; IQR: interquartile range. ^a^Chi-square test; ^b^Kruskal-Wallis Test. ^*^The definitions used for ascertaining COVID-19 vaccination status were as follows, unvaccinated: At the time of testing for SARS-CoV-2, one has not received jab or was within 14 days of first jab; partially vaccinated: did the testing for SARS-CoV-2, ≥14 days of first jab or before 14 days of second jab; fully vaccinated: did the testing for SARS-CoV-2, ≥14 days after second jab. ^**^ In post hoc analysis using adjusted standardised proportion of severe disease was significantly lower in fully vaccinated group compared to others while this was significantly higher in unvaccinated group compared to others.  ^††^In post hoc analysis using adjusted standardised proportion of severe disease was significantly lower in fully vaccinated group compared to others. [ ]: column percentage; ( ): row percentage; { }: interquartile range.
